# Immunomodulatory effects of apical papilla cells on periodontal ligament fibroblasts stimulated with *Escherichia coli* lipopolysaccharide: an *in vitro* study

**DOI:** 10.1590/1678-7757-2024-0338

**Published:** 2025-02-21

**Authors:** Alexandre Guimarães dos SANTOS, Karollyne Santos SPIGARIOL, Letícia Martins SANTOS, Marinella HOLZHAUSEN, Carla Renata SIPERT

**Affiliations:** 1 Universidade de São Paulo Faculdade de Odontologia Departamento de Dentística São Paulo SP Brasil Universidade de São Paulo, Faculdade de Odontologia, Departamento de Dentística, São Paulo, SP, Brasil.; 2 Universidade de São Paulo Faculdade de Odontologia Departamento de Biomateriais e Biologia Oral São Paulo SP Brasil Universidade de São Paulo, Faculdade de Odontologia, Departamento de Biomateriais e Biologia Oral, São Paulo, SP, Brasil.; 3 Universidade de São Paulo Faculdade de Odontologia Departamento de Estomatologia São Paulo SP Brasil Universidade de São Paulo, Faculdade de Odontologia, Divisão de Periodontia, Departamento de Estomatologia, São Paulo, SP, Brasil.

**Keywords:** Stem cells from apical papilla, Periodontal ligament fibroblasts, Inflammation

## Abstract

The role of human Stem Cells from the Apical Papilla (SCAP) in tissue regeneration has been described, but their impact on modulating the apical inflammatory process by other surrounding cell populations, such as periodontal ligament fibroblasts (PLFs), is unclear. Therefore, we investigated the role of SCAP in the activation of PLFs *in vitro*.

**Methods:**

Primary SCAP culture was used to obtain conditioned media (CM). A primary human PLF culture was established and stimulated with increasing concentrations of *Escherichia coli* lipopolysaccharide (LPS) (0.01, 0.1, and 1 µg/mL). At the 24 h time-point, an MTT viability assay was performed, and interleukin (IL)-6 and chemokine (CC-motif) ligand 2 (CCL2) levels were quantified by enzyme-linked immunosorbent assay. Then, PLFs were stimulated with LPS in the presence of SCAP-CM (1:5 dilution) for cell viability assessment and cytokine detection. The following groups were tested: PLF activated with LPS at concentrations of 0.01 and 1 µg/mL with or without SCAP-CM; a group with PLF stimulated by SCAP-CM alone; and a control group (proliferation medium only). The experiments were conducted in triplicate and sextuplicate. Statistical analyses were performed using analysis of variance followed by Tukey’s post-hoc test, with statistical significance established at 5% (p=0.05).

**Results:**

The MTT assay showed no cytotoxicity of LPS or SCAP-CM on PLFs (p>0.05). The production of CCL2 and IL-6 significantly increased in the presence of SCAP-CM regardless of the presence of LPS (p<0.0001).

**Conclusion:**

SCAP-CM significantly enhanced the release of proinflammatory cytokines by PLFs *in vitro*.

## Graphical abstract

**Figure:**
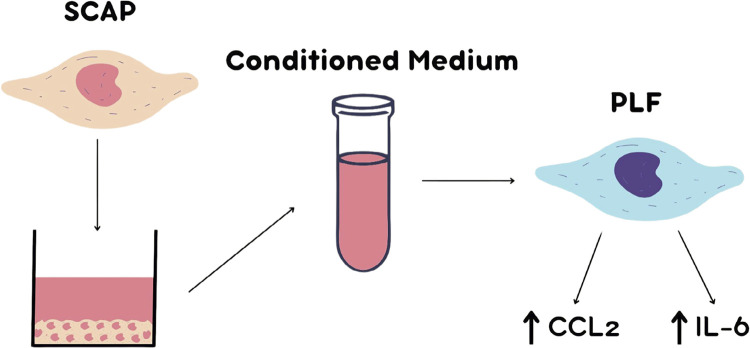

## Introduction

Apical papillae are a unique tissue associated with the roots of immature teeth, responsible for root formation.^1,2^ Stem cells of the apical papilla (SCAP) have been widely studied due to their high proliferative and differentiation capacities.^2^ This type of stem cell has been associated with the success of regenerative endodontic treatment (RET)^2^ for immature teeth, as proposed by Iwaya; Ikawa, Kubota^3^(2001), and Banchs and Trope^4^ (2004).

Many studies have attempted to describe the immunoregulatory potential of SCAP. These studies have resulted in significant findings such as their immunosuppressive capacity on microglial cells^5^ and human peripheral blood mononuclear cells.^6^ This might be attributed to the production of Transforming Growth Factor-β1 (TGF-β1), osteoprotegerin,^7^ and interleukin (IL)-10.^8^ These cells are an important secretory source of proinflammatory cytokines such as chemokine (CC-motif) ligand 2 (CCL2), substituir por: IL-6, IL-12, and tumor necrosis factor-alpha (TNF-α), with this production being accentuated in environments containing bacterial metabolites.^9,10^ Cell production of cytokines can be measured *in vitro* via different assays, such as enzyme-linked immunosorbent assay (ELISA). This method is commonly employed to analyze biomarkers, as it utilizes pre-fabricated antibodies specific to the target protein.^11^ ELISA is described as the standard for quantifying a small range of cytokines due to its superior precision when compared with other assays.^12^

The production of cytokines by SCAP might suggest their potential role on paracrine modulation of surrounding tissues and cells such as periodontal, bone, or dental pulp cells by activating their cytokines receptors. These cytokines have various roles in regulating cell proliferation, survival, and function, including inflammation. Consequently, SCAP have been employed in regenerative therapies beyond the treatment of immature teeth.^13^

The success of RET has led to its use in mature teeth with apical periodontitis,^14^ and recently in other pathologies such as external root resorption.^15^ Thus, clarifying the role of SCAP in modulating the inflammatory process resulting from root canal infection is important for understanding their potential use in the treatment of oral cavity pathologies. Since periodontal ligament fibroblasts (PLFs) are in close contact with the apical papillae during root development, understanding their paracrine modulation by SCAP is relevant. By investigating SCAP’s capacity to modulate the innate immune response of periodontal ligament cells by cytokine production, we aim to better understand their potential therapeutic role. The null hypothesis of the study was that SCAP may not significantly impact PLF modulation.

## Methodology

### Primary culture of stem cells from the apical papilla

This study was approved by the local Research Ethics Committee (Process #5.403.935). This study included human SCAP that were previously characterized. Cells were characterized according to their phenotype and function via immunostaining and flow cytometry and Alizarin Red S Staining, respectively.^7,16^ SCAP were maintained in a proliferation culture medium (PM) composed of α-Modified Eagle Medium (α-MEM) (Gibco, Waltham, MA, USA), supplemented with 10% fetal bovine serum (FBS) (Gibco), 2 mM L-glutamine (Invitrogen, Life Technologies, Carlsbad, USA), 100 µg L-ascorbate-2-phosphate (Sigma-Aldrich, St. Louis, USA), 100 U/mL penicillin, and 100 mg/ mL streptomycin (Invitrogen) at 37°C in a 5% CO_2_ atmosphere.

### Characterization of stem cells from the apical papilla

Before the experiments, SCAP were functionally characterized. Alizarin Red S Staining was performed to find the cells’ osteo/odontogenic differentiation potential. SCAP were seeded at 5×10^4^ cells per well in a 24-well plate. The proliferation and differentiation medium were replaced every two days for 14 days. Mineralization analysis was performed using 40 mmol/L Alizarin Red S Staining (Cat. A5533, Sigma-Aldrich, St. Louis, MO, USA) at pH 4.2. Calcium deposition was analyzed by adding 500 µL of ammonium hydroxide (NH_4_OH) (Êxodo, Sumaré, Brazil) to each well, and the plate was read at 405 nm in a spectrophotometer (Synergy H1, Biotek, Winooski, VT, USA). Additionally, the colony-forming unit assay was performed to analyze SCAP proliferation potential. Cells were plated in six-well plates at a density of 100 cells per well, in triplicate, and incubated for seven days. The proliferation medium (α-MEM with 10% FBS) was changed every two days. Cells were then fixed using Rapid Hematology Stains (RenyLab, Barbacena, Brazil) and observed under an optical microscope.

### Preparation of SCAP-conditioned medium (SCAP-CM)

Cells at 90% confluency were detached with 0.25% trypsin EDTA (Invitrogen), seeded into a 6-well plate at a density of 2×10^5^ cells, and incubated in α-MEM with 10% FBS for 24 h at 37°C in a 5% CO_2_ atmosphere. Subsequently, the medium was replaced every 24 h for two days with FBS-free α-MEM and further incubated at 37°C in 5% CO_2_. The supernatants were collected, centrifuged at 800 × *g* for 10 min at 4°C, and stored at −80°C.

### Periodontal ligament fibroblasts (PLF) culture and LPS Cytotoxicity assay

The PLF was cultured following the exact parameters of SCAP culture. Cells were seeded at a density of 2 × 10^4^ cells/cm^2^ in 24-well plates and incubated in α-MEM with 10% FBS for 24 h. The medium was then replaced with serum-free α-MEM and incubated for an additional 24 h. Subsequently, cells were stimulated with *Escherichia coli* LPS (Cat. L6529, Sigma-Aldrich) at concentrations ranging from 0.01 to 1 µg/mL. Supernatants were collected after 24 h at serum-free medium, centrifuged at 800 × *g* for 10 min at 4°C, and stored at −80°C for future cytokine quantification analysis. After that, the cells were subjected to an MTT (3-[4,5-dimethylthiazol-2-yl]-2,5-diphenyltetrazolium bromide) (Invitrogen) assay to analyze LPS cytotoxicity on PLF. Each well was treated with 40 µL of MTT solution (5 mg/mL in PBS) and 360 µL of 10% FBS α-MEM. Cells were then incubated for 4 h at 37°C in a 5% CO_2_ atmosphere. Then, the solution was discarded, and 200 µL of DMSO (dimethyl sulfoxide) (Labsynth, Diadema, Brazil) was added to each well. The absorbance was measured using a spectrophotometer at a wavelength of 570 nm.

### LPS-activation of PLF under SCAP-CM

PLF were seeded into 24-well plates at a density of 2×10^4^ cells/cm^2^ and incubated for 24 h. The medium was then replaced with serum-free α-MEM for an additional 24 h. Subsequently, PLF were stimulated with LPS (0.01 and 1 µg/mL) in the presence of SCAP-CM (diluted 1:5). An MTT assay was performed after 48 h, and supernatants were collected for cytokine detection.

### Enzyme-Linked Immunosorbent Assay

The cytokines IL-6 and CCL2 were quantified from PLF supernatants in both experimental sets. Commercial kits (DuoSet enzyme-linked immunosorbent assay kits, DY279-05 and DY206-05, R&D Systems, Minneapolis, MN, USA) were employed for detecting soluble proteins following the manufacturer’s instructions. In brief, a 96-well plate was coated with anti-human IL-6, or CCL2 monoclonal antibodies, and incubated overnight, protected from the light. Then, the blocking solution (phosphate-buffered saline with 1% of bovine serum albumin - PBS BSA 1%) was added to each well and incubated for 2 h. Next, a standard curve was prepared with serially diluted (1/2) recombinant human IL-6, or CCL2, and incubated, as well as the cell supernatant’s (samples). After 2 h, biotinylated antibodies were placed in each well and incubated for additional 2 h. Afterwards, horseradish peroxidase was added and incubated followed by a substrate solution that was further incubated for the same time (20 min). Finally, the reaction was stopped by the addition of 2N sulfuric acid, and the plates were read in a spectrophotometer at a wavelength of 490 nm. For control purposes, cell-free wells (SCAP-CM only) were tested for both cytokines. The experiment was conducted in triplicate.

### Experimental design

In summary, at the first experiment set, LPS cytotoxicity and the production of the cytokines CCL2 and IL-6 were assessed at LPS-activated PLFs (0.01, 0.1, and 1 µg/mL) and a control group (proliferation medium only). At the second experiment set, PLFs were subjected to LPS stimulation (0.01 and 1 µg/mL) at the presence of SCAP-CM. In total, six experimental conditions were defined: (1): PLFs at medium only (control group); (2): PLFs + SCAP-CM; (3): PLFs + 0.01 µg/mL LPS; (4): PLFs + 0.01 µg/mL LPS + SCAP-CM; (5): PLFs + 1 µg/mL LPS; (6): PLFs + 1 µg/mL + SCAP-CM. SCAP-CM only was kept at cell-free wells as an internal control for cytokine detection. All experiments were conducted in triplicate.^9,17^

### Statistical analysis

The normality of data was assessed using Shapiro-Wilk test. To analyze SCAP calcium deposition, data analysis was conducted using Unpaired t test. The next analyses were performed using either one-way analysis of variance (ANOVA) followed by Tukey’s post-test when LPS was the only variable studied, or two-way ANOVA followed by Tukey’s post-test when two variables were analyzed (LPS concentration and SCAP-CM). Significance was set at p<0.05. Data analysis was performed using GraphPad Prism 9.0 (GraphPad Software, San Diego, CA, USA).

## Results

### Characterization of SCAP

**Figure 1:**
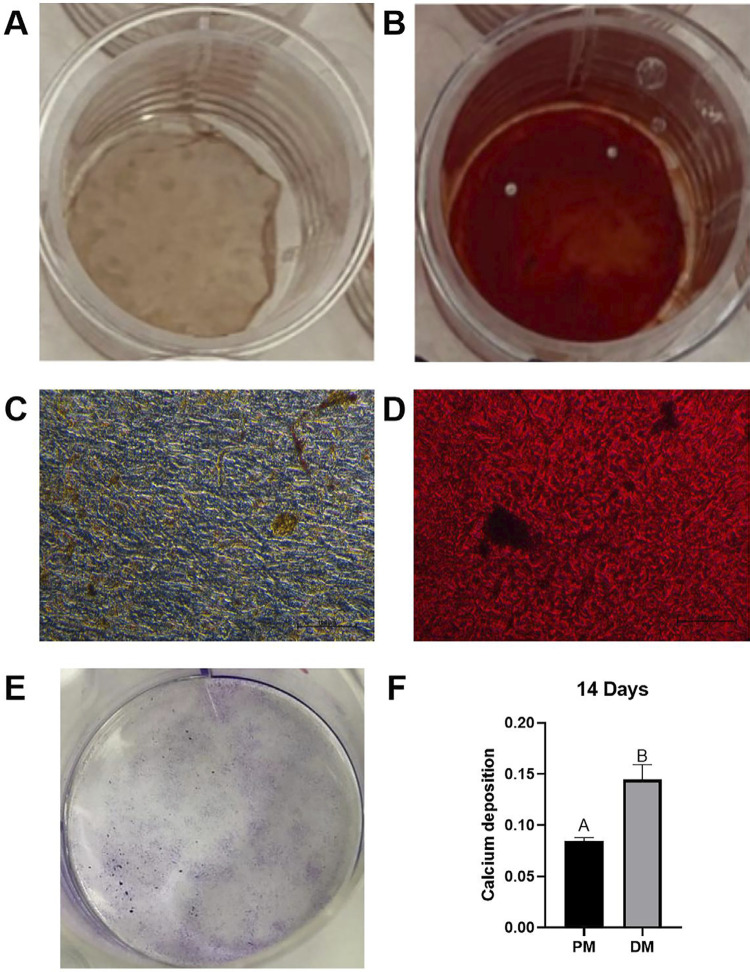
Functional characterization of cultured SCAP. Mineralization potential of Stem Cells of the Apical Papilla induced by differentiation for 14 days detected by Alizarin Red S Staining. Macroscopic (A and B) and microscopic (C and D) view of cells under proliferation (A and C) or differentiation medium (B and D). Macroscopic view of colony forming unit assay: cells fixed by rapid hematology stains (E). Densitometric analysis of calcium deposition by SCAP kept at proliferation medium (PM) or differentiation medium (DM) after 14 days: different letters represent statistical differences between groups (simple t-test unpaired, p<0.05, N=3) (F).

The osteo/odontogenic differentiation potential of SCAP was confirmed by detecting calcium deposition via Alizarin Red S Staining (Figure 1A-D). Semi-analytical densitometry revealed a statistically significant difference (p=0.0020) between the proliferation medium (mean = 0.08467±0.031) and the differentiation medium (mean = 0.1447±0.0142) (Figure 1F). Moreover, the proliferative potential of SCAPs was demonstrated using a colony-forming unit assay (Figure 1E).

### Cytotoxicity and cytokines production by LPS-treated PLFs

**Figure 2:**
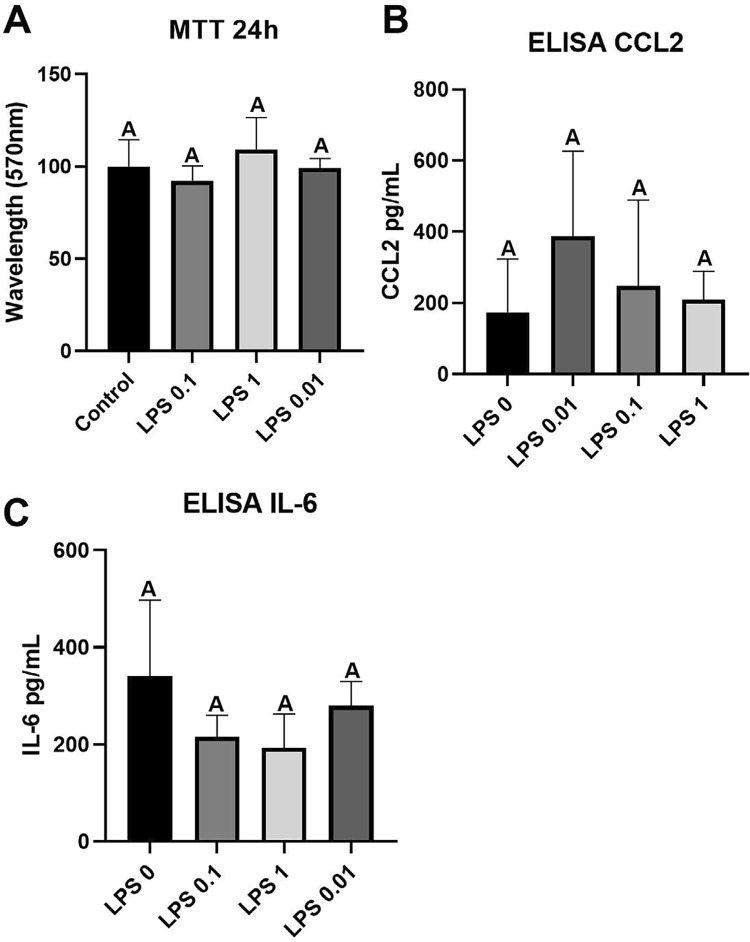
Cytotoxicity and cytokines production by LPS-activated PLF. Absorbance (570nm) data obtained from the MTT cellular viability assay at 24 h time point in PLF activated with LPS (0; 0.01, 0.1, and 1 µg/mL) (A). Production of CCL2 (B) and IL-6 (C) by PLF according to the ELISA assay after 24 h of exposure to different concentrations of E. Coli LPS (0; 0.01, 0.1, and 1 µg/mL). Results represented as means and standard deviations (n=6). Different letters represent statistical differences between groups (One-Way ANOVA with Tukey’s test, p<0.05).

The MTT assay was performed to estimate cytotoxic concentrations of LPS. The results showed that none of the analyzed LPS concentrations (0.01, 0.1, and 1 µg/mL) compromise the viability of PLFs (p=0.1676) (Figure 2A). In addition, none of the tested LPS concentrations altered constitutive CCL2 (mean = 257.2±66.34) (Figure 2B) and IL-6 (mean = 255±81.39) (Figure 2C) levels (p=0.3472 and 0.076, respectively). Based on these findings, the concentrations of 0.01 and 1 µg/mL were chosen for subsequent experiments.

### Cytotoxicity and cytokines production by PLFs treated simultaneously with LPS and SCAP-CM

**Figure 3:**
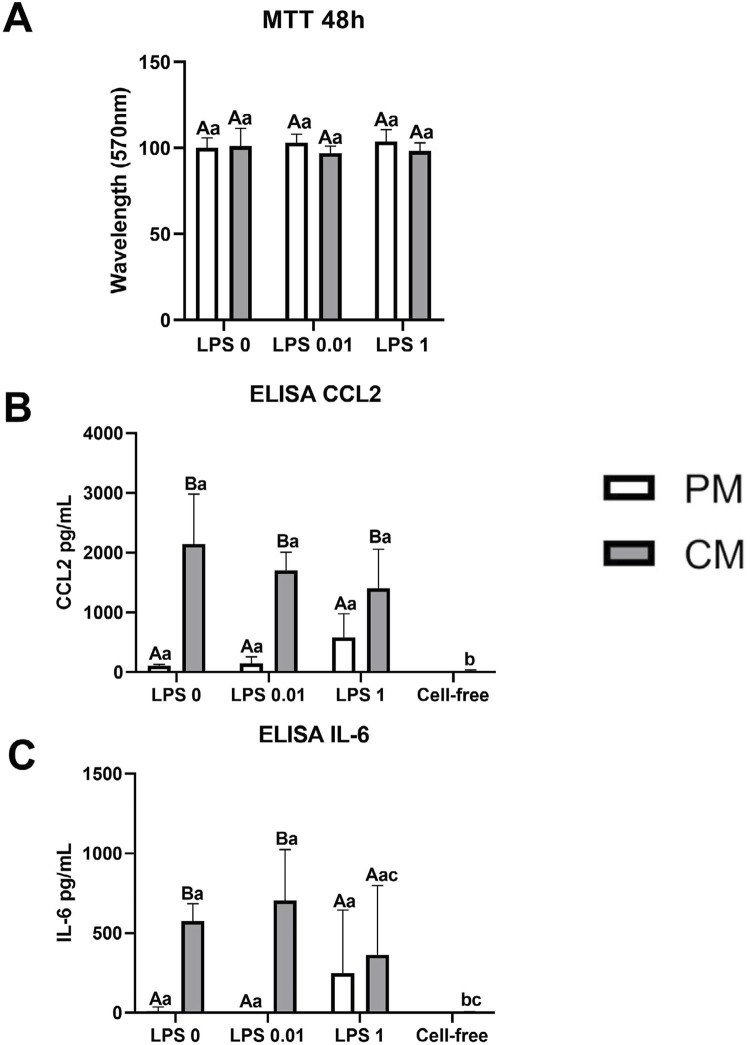
Cytotoxicity and cytokines production by PLF simultaneously LPS and SCAP conditioned medium activation. Absorbance (570 nm) data obtained from the MTT cellular viability assay at 48 h time point in PLF activated with LPS (0; 0.01, and 1 µg/mL) stimulated or not with SCAP conditioned medium (1/5) (A). Production of CCL2 (B) and IL-6 (C) by PLF according to the ELISA assay after 24 h of exposure to different concentrations of E. Coli LPS (0; 0.01, and 1 µg/mL) and under proliferation medium (PM) or SCAP conditioned medium (CM) (1/5). Results represented as means and standard deviations of the experiments performed in sextuplicate. Different capital letters represent statistical differences between groups at the same LPS concentration. Different lowercase letters represent statistical differences between groups of different LPS concentrations (Two-Way ANOVA with Tukey’s test, p<0.05).

Neither SCAP-CM nor LPS altered the viability of PLFs at the 48-h time point, as shown by the MTT assay (mean = 100.54±2.404. p>0.1406) (Figure 3A). Interestingly, the presence of SCAP-CM resulted in a significant increase in both CCL2 (mean = 1752.333±306.657) (Figure 3B) and IL-6 (mean = 547.7±140.39) (Figure 3C) production by PLFs compared to the respective controls, regardless of the presence of LPS (p<0.0001). These cytokines were not detected in cell-free wells containing SCAP-CM alone (Figure 3B and C).

## Discussion

SCAP have been studied by the research community due to their great potential in regeneration treatments. This potential can be attributed to SCAP’s proliferation and immunoregulatory effects.^2^ For example, SCAP contribute to tissue neoformation in RET by facilitating the formation of a blood clot that fills the root canal.^2^ CCL2 and IL-6 are responsible for monocyte recruitment and the inhibition of neutrophil apoptosis, respectively.^18,19^ Consequently, studying the potential of SCAP to modulate PLFs is fundamental since these two cell populations are in close contact during root formation, and the presence of proinflammatory cytokines can modulate phagocytes.^20^ This, in turn, might contribute to oral resorptive diseases, such as bone resorption at apical periodontitis.^21^

In the context of oral health, monocytes play a crucial role as phagocytes, engulfing microorganisms and dead cells, being essential for the body’s defense. They are mainly recruited via CCL2,^18^ therefore, an increased local concentration of this chemokine will result in a higher defense potential by the immune system. However, monocytes are also precursors of osteoclasts that are involved in bone physiological remodeling and oral diseases such as apical periodontitis.^22^ In a scenario of chronic inflammation, the balance between bone formation and resorption is broken, resulting in bone loss.^23^ This ambiguous action of CCL2 depends on the presence of other metabolites in the inflamed tissue such as macrophage colony-stimulating factor (M-CSF) and receptor activator of nuclear factor kappa-Β ligand (RANKL), that in turn will differentiate monocytes into osteoclasts.^22^ Therefore, the increase in the local concentration of CCL2 via SCAP increases monocyte migration, and might improve macrophage and osteoclast differentiation.

IL-6 is a proinflammatory cytokine responsible for inducing the production of proinflammatory proteins, including C-reactive protein and fibrinogen.^24^ It also plays a role in antibody production and differentiation of T lymphocytes.^25^ In addition, once present in the periodontal ligament environment, IL-6 can inhibit neutrophil apoptosis.^19^ IL-6 is also important for phagocyte function and provokes osteoclast differentiation by inducing the expression of RANKL.^26^ Consequently, the increase of IL-6 contributes to periodontal or apical bone loss.

Overall, the results of our study demonstrated that both IL-6 and CCL2 are produced constitutively by PLFs *in vitro*, and LPS does not significantly impact their production (Figure 2B and C). Surprisingly, the production of CCL2 and IL-6 by PLFs was significantly increased under SCAP-CM stimulation irrespective of the presence of LPS. These findings cannot be attributed to the possible presence of these cytokines in the SCAP supernatant since cell-free wells, containing SCAP-CM only, were tested as control under the same experimental conditions and did not present detectable amounts of these cytokines (Figure 3B and C).

These results suggest that apical papilla cells may enhance the activation of PLFs, regardless of the presence of bacterial byproducts such as LPS, a component of Gram-negative bacteria that are commonly found in the root canal after pulp necrosis.^27,28^ For this reason, LPS was selected as the inflammatory activator in our study.

Periodontal ligament stem cells are positively regulated by CCL2^29^ and IL-6, as shown by the receptor IL6R expression.^30^ Additionally, it has been demonstrated that SCAP shows a significant potential to modulate inflammation and the secretion of IL-6 and CCL2.^9,10^ Our results clarifies the paracrine activation of PLF by SCAP. Specifically, we found an increased production of both IL-6 and CCL2 in PLFs in the presence of SCAP-CM, regardless of the presence of bacterial byproducts. This finding may also be related to the potential of some other metabolites from SCAP to activate periodontal ligament cells, such as IL-8,^31^ which regulates angiogenesis, induces the migration of neutrophils^(32)^, and activates tissue inhibitors of metalloproteinases-1,^31^ a factor involved in periodontal remodeling.^33^

Regenerative treatments have been extensively studied in the Dentistry and Medicine fields in recent years. These types of procedures need three main pillars to succeed: residual stem cells, scaffold, and growth factors.^34,35^ SCAP is considered one of the most important stem cells involved in RET due to their high proliferative and differentiation potential.^2,31^ However, we should not underestimate the possibility of migrating periodontal cells into the root canal during apical bleeding induction^36^ and also their potential role in inflammation establishment in response to root canal remaining bacterial byproducts. Additionally, the use of SCAP in clinical practice is still restricted to RET; however, they might be further considered for periodontal reconstruction *in vivo*.^1,13^

The culture of human SCAP was first established and characterized by Sonoyama, et al.^1^ (2006). This cell population is recognized as the primary source of odontoblasts and exhibits enhanced proliferation and differentiation potential compared to dental pulp stem cells.^2^ Consequently, numerous studies have employed SCAP cultures to investigate the mechanisms involved in cell differentiation, evaluate the biocompatibility of chemical substances used in revascularization, and understand the role of pathogens in the failure of regenerative endodontics.^37,38^ To date, this cell population is considered a valuable tool for research in these areas.

The use of cell-conditioned media in *in vitro* assays with various cell cultures provides an alternative to traditional co-culture experiments. These assays enable the study of the paracrine effects of molecules released by one cell population on others, such as cells from the apical papilla and periodontal ligament.^39^ However, as an *in vitro* study, certain limitations must be considered. PLF were evaluated in the presence of LPS and SCAP-CM; however, from a clinical perspective, additional metabolites from bacteria and surrounding cells would likely interact with periodontal ligament cells, potentially altering the results. Therefore, the results should be interpreted with caution, as they do not fully replicate the complexity of biological phenomena. Future research could involve stimulating PLF with intracanal biofilm to provide a more comprehensive bacterial substrate, potentially enhancing the findings. Additionally, analyzing a broader range of cytokines could provide a more expansive understanding of SCAP paracrine modulation.

In spite of the limitations of the study, we speculate that, during root formation, the immune response, along with the activation of PLFs, might be enhanced due to the presence of the apical papilla, potentially leading to more robust phagocyte recruitment compared to what occurs in mature teeth. These findings might also contribute to regenerative endodontic procedures, as periodontal ligament cells might also contribute to the inflammatory response against remaining microorganisms.

## Conclusion

The results of this study showed that SCAP increased the levels of proinflammatory cytokines produced by PLF *in vitro*. Thus, these findings could provide new insights into the use of SCAP for therapeutic interventions in oral pathologies. The null hypothesis of the study was rejected.
